# Supplementary material for: Reduction in Spoilage Microbiota and Cyclopiazonic Acid Mycotoxin with Chestnut Extract Enriched Chitosan Packaging: Stability of Inoculated Gouda Cheese
Source: Foods. 2020 Nov 11;9(11):1645. doi: 10.3390/foods9111645 (PMC7697305; doi:10.3390/foods9111645)
Supplement: Supplementary file 1 [file foods-09-01645-s001.pdf]

foods

Supplementary data

Reduction in Spoilage Microbiota and Cyclopiazonic Acid Mycotoxin with Chestnut Extract Enriched Chitosan Packaging: Stability of Inoculated Gouda Cheese

Kristi Kõrge [1,2], Helena Šeme [3], Marijan Bajić [1], Blaž Likozar [1], Uroš Novak [1,*]

[1] Department of Catalysis and Chemical Reaction Engineering, National Institute of Chemistry, Hajdrihova 19, 1000 Ljubljana, Slovenia; kristi.korge@ki.si (K.K.); marijan.bajic@ki.si (M.B.); blaz.likozar@ki.si (B.L.)

[2] Department of Chemistry and Biotechnology, Tallinn University of Technology, Akadeemia tee 15, 12618 Tallinn, Estonia

[3] Acies Bio d.o.o., Tehnološki park 21, 1000 Ljubljana, Slovenia; helena.seme@aciesbio.com

* Correspondence: uros.novak@ki.si

1. Mechanical properties of the films

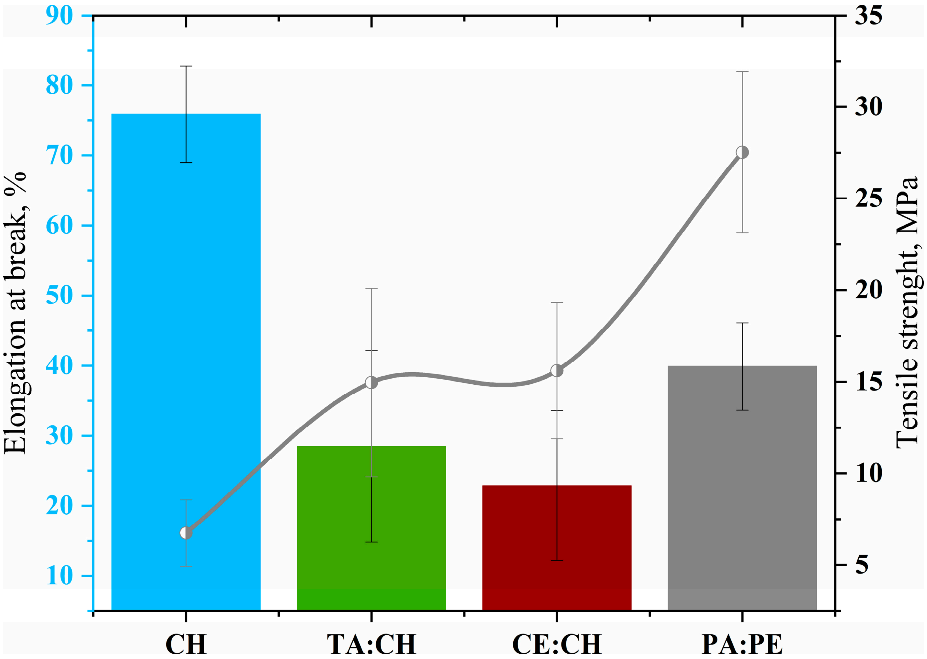

Figure S1. Mechanical properties of films used for packing Gouda cheese. Values are means (n = 4 × 3) with standard errors that are significantly different within columns ($p < 0.05$).

2. Activity of the films

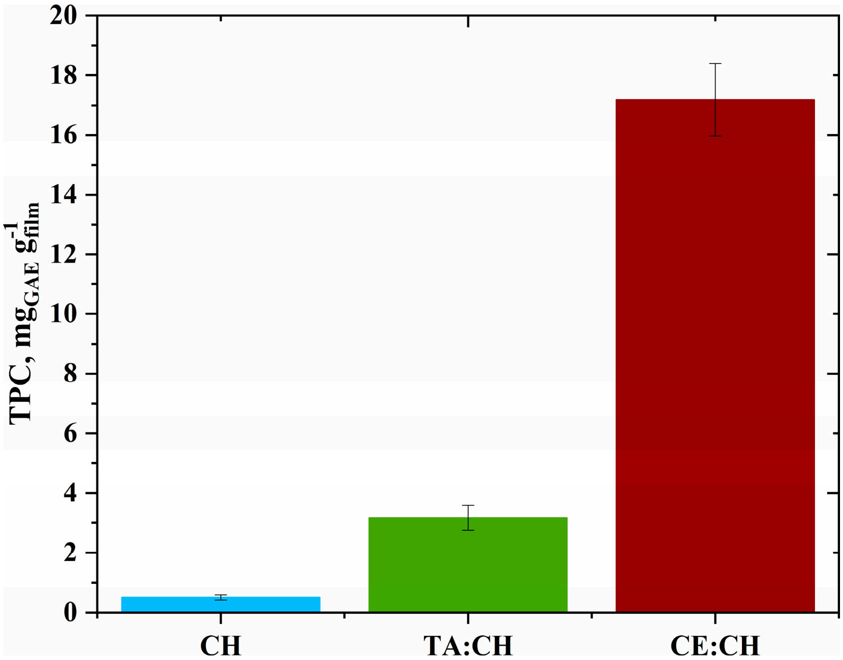

Figure S2. Total phenolic content of films used for packing Gouda cheese. Gallic acid was used as a standard, and the results were expressed as the mass of gallic acid equivalent (GAE) per mass of the film. Values are means (n = 2 × 3) with standard errors that are significantly different within columns ($p < 0.05$).

3. Bacteria and fungi reduction with chitosan-based films

Table S1. The effect of biopolymer film (CH, TA:CH, CE:CH) on CPA production in cheese (0.5 g), compared with mycotoxin accumulation in films (2 × 2 cm) during storage at 4 °C and 25 °C. Slash-marked spaces depict missing parallels for CH and CH:CE samples and unable to record results for TA:CH and iTA:CH. Calculations in µg/kg are expressed per 1 g of the sample. Values are means (n = 2 × 3) with standard errors that are significantly different within rows ($p < 0.05$).

E. coli 4 °C

Sample	0. day	7. day	14. day	37. day
iCHE CH	6.54 ± 0.59	5.94 ± 0.14	4.90 ± 0.23	4.63 ± 0.12
iCHE TA		5.63 ± 0.32	5.58 ± 0.31	5.73 ± 0.21
iCHE CE		5.34 ± 0.13	5.17 ± 0.19	5.55 ± 0.21
iCHE PA:PE		6.74 ± 0.08	6.55 ± 0.20	6.57 ± 0.07

P. fluorescens 4 °C

Sample	0. day	7. day	14. day	37. day
iCHE CH	8.30 ± 0.09	7.30 ± 0.27	6.51 ± 0.19	2.26 ± 0.25
iCHE TA		7.50 ± 0.46	7.00 ± 0.08	4.05 ± 0.19
iCHE CE		7.51 ± 0.34	6.79 ± 0.08	4.33 ± 0.24
iCHE PA:PE		8.13 ± 0.50	7.38 ± 0.99	5.59 ± 0.07

P. commune 4 °C

Sample	0. day	7. day	14. day	37. day
iCHE CH	4.35 ± 0.42	3.65 ± 0.38	3.85 ± 0.06	3.51 ± 0.12
iCHE TA		3.68 ± 0.27	3.35 ± 0.15	3.29 ± 0.13
iCHE CE		3.73 ± 0.27	3.65 ± 0.09	3.61 ± 0.10

iCHE PA:PE		3.86 ± 0.30	3.74 ± 0.11	3.88 ± 0.23
P. commune 25 °C				
Sample	0. day	7. day	14. day	37. day
iCHE CH	4.23 ± 0.32	3.68 ± 0.37	3.40 ± 0.18	3.26 ± 0.16
iCHE TA		3.59 ± 0.33	3.47 ± 0.12	2.41 ± 0.27
iCHE CE		3.74 ± 0.29	3.51 ± 0.27	2.56 ± 0.33
iCHE PA:PE		3.83 ± 0.48	3.72 ± 0.07	2.70 ± 0.18
Blank 4 °C and Blank 25 °C				
Sample	0. day	7. day	14. day	37. day
iCHE CH				
iCHE TA		Below detection		
I CHE CE				
iPA:PE				

foods

MDPI

4. Influence of chitosan-based films on mycotoxin CPA from cheese

Table S2. The effect of biopolymer film (CH, TA:CH, CE:CH) on CPA production in cheese (0.5 g), compared with mycotoxin accumulation in films (2 × 2 cm) during storage at 4 °C and 25 °C. Slash marked spaces mark missing parallels for CH and CH:CE samples and unable to record results for TA:CH and iTA:CH. Calculations in µg/kg are expressed per 1 g of the sample. Values are gained by subtraction of spiked noninoculated sample mean from the spiked inoculated sample mean values, that are significantly different within rows ($p < 0.05$).

**Time**		0. day		7. day		14. day		37. day	
**Sample**		mg/mL	µg/kg	mg/mL	µg/kg	mg/mL	µg/kg	mg/mL	µg/kg
iCHE PA:PE	4 °C	0.0084 ± 0.0001	33467 ± 503	0.0088 ± 0.0001	35000 ± 283	0.0152 ± 0.0004	60700 ± 1740	0.0220 ± 0.0001	87867 ± 462
CHE PA:PE				0.0084 ± 0.0001	33600 ± 566	0.0145 ± 0.0015	57800 ± 6044	0.0219 ± 0.0001	87600 ± 566
iCHE CH				0.0082 ± 0.0003	32600 ± 1058	0.0151 ± 0.0004	60200 ± 1405	0.0240 ± 0.0005	95800 ± 2104
CHE CH				0.0080 ± 0.0000	31900 ± 200	0.0147 ± 0.0005	58800 ± 2078	0.0225 ± 0.0006	90000 ± 2286
iCHE TA				0.0092 ± 0.0002	36800 ± 924	0.0164 ± 0.0011	65467 ± 4388	0.0298 ± 0.0006	119200 ± 2422
CHE TA				0.0087 ± 0.0002	34933 ± 611	0.0163 ± 0.0002	65200 ± 693	0.0279 ± 0.0012	111600 ± 4607
iCHE CE				0.0119 ± 0.0002	47600 ± 980	0.0199 ± 0.0024	79600 ± 9708	0.0436 ± 0.0035	174267 ± 14110
CHE CE				0.0104 ± 0.0003	41700 ± 1000	0.0194 ± 0.0002	77700 ± 683	0.0415 ± 0.0020	165900 ± 8002
iCHE PA:PE	25 °C	0.0084 ± 0.0001	33467 ± 503	0.0075 ± 0.0001	30133 ± 231	0.0131 ± 0.0001	52200 ± 231	0.0258 ± 0.0023	103200 ± 9304
CHE PA:PE				0.0073 ± 0.0001	29333 ± 462	0.0130 ± 0.0001	52133 ± 231	0.0255 ± 0.0001	101800 ± 283
iCHE CH				0.0076 ± 0.0000	30400 ± 0	0.0130 ± 0.0001	52000 ± 327	0.0277 ± 0.0001	110900 ± 383
CHE CH				0.0076 ± 0.0001	30200 ± 516	0.0130 ± 0.0002	51800 ± 849	0.0277 ± 0.0004	110700 ± 1612
iCHE TA				0.0092 ± 0.0004	36600 ± 1774	0.0153 ± 0.0002	61000 ± 958	0.0304 ± 0.0004	121700 ± 1400
CHE TA				0.0083 ± 0.0003	33000 ± 1200	0.0141 ± 0.0000	56300 ± 200	0.0301 ± 0.0001	120400 ± 566
iCHE CE				0.0127 ± 0.0000	50800 ± 0	0.0188 ± 0.0002	75200 ± 800	0.0367 ± 0.0014	146933 ± 5787
CHE CE				0.0111 ± 0.0009	44400 ± 3464	0.0183 ± 0.0002	73200 ± 800	0.0366 ± 0.0003	146400 ± 1131
iCH	4 °C	0.0100 ± 0.0002	39800 ± 849	0.0092 ± 0.0002	36900 ± 825	0.0125 ± 0.0001	50133 ±231	0.0262 ± 0.0007	104600 ± 2723
CH				/	/	0.0121 ± 0.0001	48400 ± 400	0.0263 ± 0.0002	105200 ± 800

iCH:TA				/	/	/	/	/	/
CH:TA				/	/	/	/	/	/
iCH:CE		0.0107 ± 0.0001	42600 ± 283	0.0153 ± 0.0011	61000 ± 4555	0.0199 ± 0.0002	79400 ± 849	0.0303 ± 0.0020	121000 ± 8017
CH:CE				/	/	0.0191 ± 0.0001	76200 ± 283	0.0244 ± 0.0007	133700 ± 2740
iCH	25 °C	0.0100 ± 0.0002	39800 ± 849	0.0084 ± 0.0002	33700 ± 683	0.0121 ± 0.0002	48533 ± 833	0.0231 ± 0.0001	92500 ± 503
CH				0.0087 ± 0.0002	34933 ± 611	0.0120 ± 0.0002	47867 ± 833	0.0226 ± 0.0041	90300 ± 16390
iCH:TA				/	/	/	/	/	/
CH:TA				/	/	/	/	/	/
iCH:CE		0.0107 ± 0.0001	42600 ± 283	0.0110 ± 0.0002	44100 ± 887	0.0141 ± 0.0003	56400 ± 1131	0.0250 ± 0.0029	100133 ± 11547
CH:CE				0.0109 ± 0.0006	43400 ± 2546	0.0134 ± 0.0005	53467 ± 1973	0.0242 ± 0.0022	96933 ± 8812

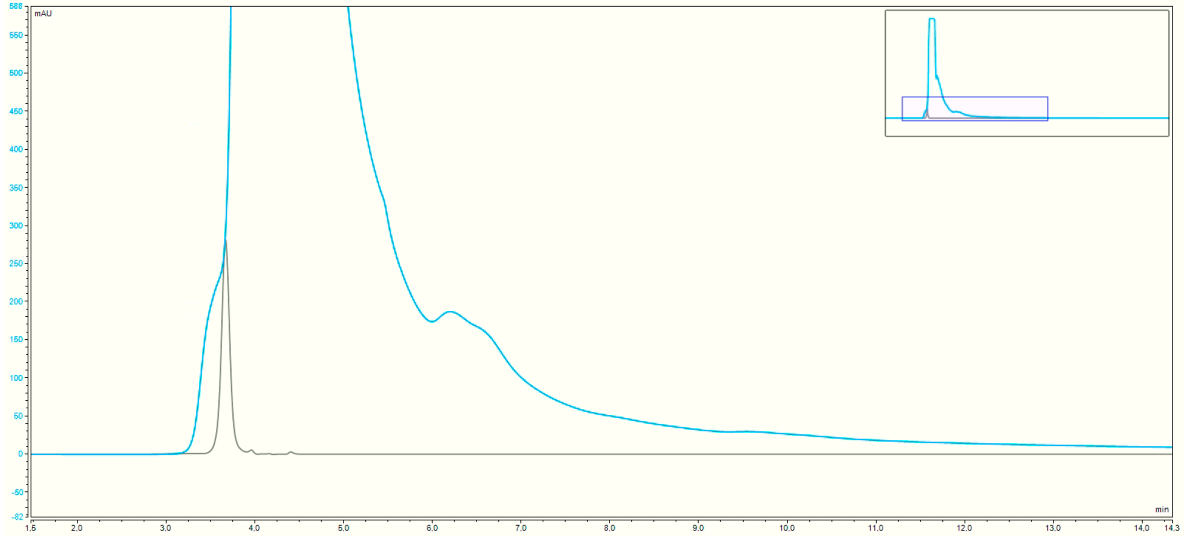

Figure S3. UHPLC chromatograms of iTA:CH (film) inoculated with *P. commune* (■) and 0.025 mg/L CPA standard (■). Spectre depicts possible shadowing complex formation between the CPA- and TA- containing samples.

5. pH value

Table S3. The effect of biopolymer film (CH, TA:CH, CE:CH) on pH in cheese, compared to simultaneous pH change in films (2 × 2 cm) at 4 °C and 25 °C. Values are means (n = 2 × 1) with standard errors that are significantly different within columns ($p < 0.05$).

a *Blank 4 °C*

	CHE CH	CHE TA	CHE CE	CHE PA:PE	CH	TA:CH	CE:CH
0. day	5.59 ± 0.06	5.59 ± 0.06	5.59 ± 0.06	5.59 ± 0.06	3.89 ± 0.04	3.80 ± 0.01	3.19 ± 0.03
7. day	5.50 ± 0.03	5.59 ± 0.04	5.43 ± 0.06	5.88 ± 0.02	/	/	/
14. day	5.35 ± 0.05	5.42 ± 0.00	5.40 ± 0.03	5.80 ± 0.02	4.79 ± 0.01	4.81 ± 0.00	4.41 ± 0.25
37. day	5.32 ± 0.00	5.39 ± 0.05	5.34 ± 0.00	5.75 ± 0.03	4.83 ± 0.00	4.77 ± 0.03	4.50 ± 0.17

b *Blank 25 °C*

	CHE CH	CHE TA	CHE CE	CHE PA:PE	CH	TA:CH	CE:CH
0. day	5.59 ± 0.06	5.59 ± 0.06	5.59 ± 0.06	5.59 ± 0.06	3.89 ± 0.04	3.80 ± 0.01	3.19 ± 0.03
7. day	5.42 ± 0.01	5.49 ± 0.00	5.48 ± 0.03	5.81 ± 0.03	4.87 ± 0.05	4.87 ± 0.01	4.55 ± 0.19
14. day	5.34 ± 0.03	5.34 ± 0.05	5.33 ± 0.01	5.75 ± 0.04	4.99 ± 0.04	4.85 ± 0.11	4.80 ± 0.00
37. day	5.23 ± 0.02	5.27 ± 0.07	5.24 ± 0.02	5.89 ± 0.03	5.01 ± 0.04	4.90 ± 0.13	4.74 ± 0.08

c *P. commune 25 °C*

	CHE CH	CHE TA	CHE CE	CHE PA:PE	CH	TA:CH	CE:CH
0. day	5.59 ± 0.06	5.59 ± 0.06	5.59 ± 0.06	5.59 ± 0.06	3.89 ± 0.04	3.80 ± 0.01	3.19 ± 0.03
7. day	5.43 ± 0.02	5.51 ± 0.01	5.46 ± 0.07	5.87 ± 0.06	5.12 ± 0.01	4.52 ± 0.00	4.58 ± 0.09
14. day	5.36 ± 0.03	5.39 ± 0.01	5.32 ± 0.00	5.70 ± 0.03	5.04 ± 0.07	4.88 ± 0.07	4.74 ± 0.06
37. day	5.30 ± 0.02	5.27 ± 0.02	5.24 ± 0.02	5.88 ± 0.05	4.99 ± 0.01	4.27 ± 0.03	4.78 ± 0.01

d *P. fluorescens 4 °C*

	CHE CH	CHE TA	CHE CE	CHE PA:PE	CH	TA:CH	CE:CH
0. day	5.59 ± 0.06	5.59 ± 0.06	5.59 ± 0.06	5.59 ± 0.06	3.89 ± 0.04	3.80 ± 0.01	3.19 ± 0.03
7. day	5.45 ± 0.01	5.65 ± 0.09	5.46 ± 0.02	6.02 ± 0.08	/	/	/

14. day	5.33 ± 0.01	5.51 ± 0.08	5.41 ± 0.06	5.75 ± 0.02	4.95 ± 0.06	4.73 ± 0.11	4.59 ± 0.01
37. day	5.29 ± 0.01	5.34 ± 0.02	5.21 ± 0.04	5.77 ± 0.02	4.91 ± 0.05	4.72 ± 0.13	4.62 ± 0.05

e				*E. coli 4 °C*			
	CHE CH	CHE TA	CHE CE	CHE PA:PE	CH	TA:CH	CE:CH
0. day	5.59 ± 0.06	5.59 ± 0.06	5.59 ± 0.06	5.59 ± 0.06	3.89 ± 0.04	3.80 ± 0.01	3.19 ± 0.03
7. day	5.58 ± 0.06	5.59 ± 0.00	5.51 ± 0.03	5.96 ± 0.26	/	/	/
14. day	5.36 ± 0.06	5.57 ± 0.01	5.40 ± 0.03	5.84 ± 0.03	4.84 ± 0.07	4.82 ± 0.08	4.52 ± 0.02
37. day	5.23 ± 0.04	5.35 ± 0.01	5.27 ± 0.02	5.81 ± 0.00	4.95 ± 0.00	4.82 ± 0.01	4.56 ± 0.09

f				*P. commune 4 °C*			
	CHE CH	CHE TA	CHE CE	CHE PA:PE	CH	TA:CH	CE:CH
0. day	5.59 ± 0.06	5.59 ± 0.06	5.59 ± 0.06	5.59 ± 0.06	3.89 ± 0.04	3.80 ± 0.01	3.19 ± 0.03
7. day	5.41 ± 0.00	5.51 ± 0.03	5.48 ± 0.03	5.83 ± 0.06	4.83 ± 0.01	4.28 ± 0.02	4.01 ± 0.13
14. day	5.35 ± 0.04	5.47 ± 0.00	5.42 ± 0.02	5.75 ± 0.05	5.00 ± 0.09	4.88 ± 0.11	4.44 ± 0.10
37. day	5.29 ± 0.02	5.40 ± 0.01	5.33 ± 0.01	5.77 ± 0.08	4.98 ± 0.06	4.76 ± 0.05	4.45 ± 0.08

6. Sensorial perception of the cheese packed in chitosan-based films

Visual appearances of the Gouda cheese samples were captured to describe potential sensory perception and quality of the contaminated product when packed in chitosan film packaging (Figure 5). It is possible to see how both the packaging itself and the Gouda cheese looked like in the packaging set at the beginning of the study (Figure S4, S5).

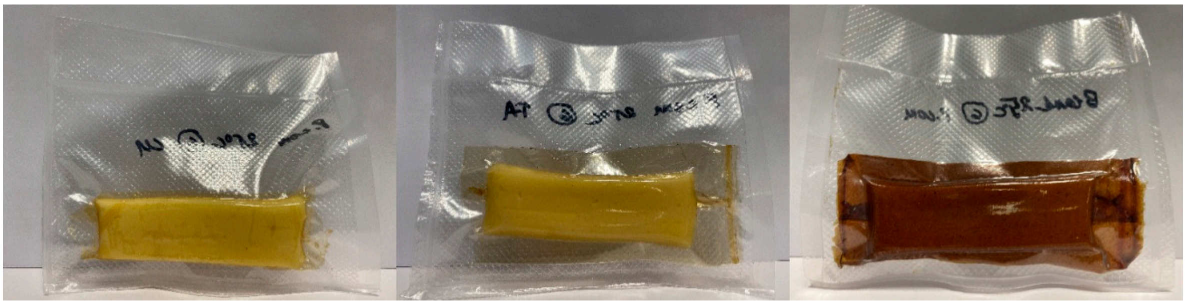

Figure S4. Gouda cheese packed in CH, TA:CH and CE:CH biofilms (from left to right).

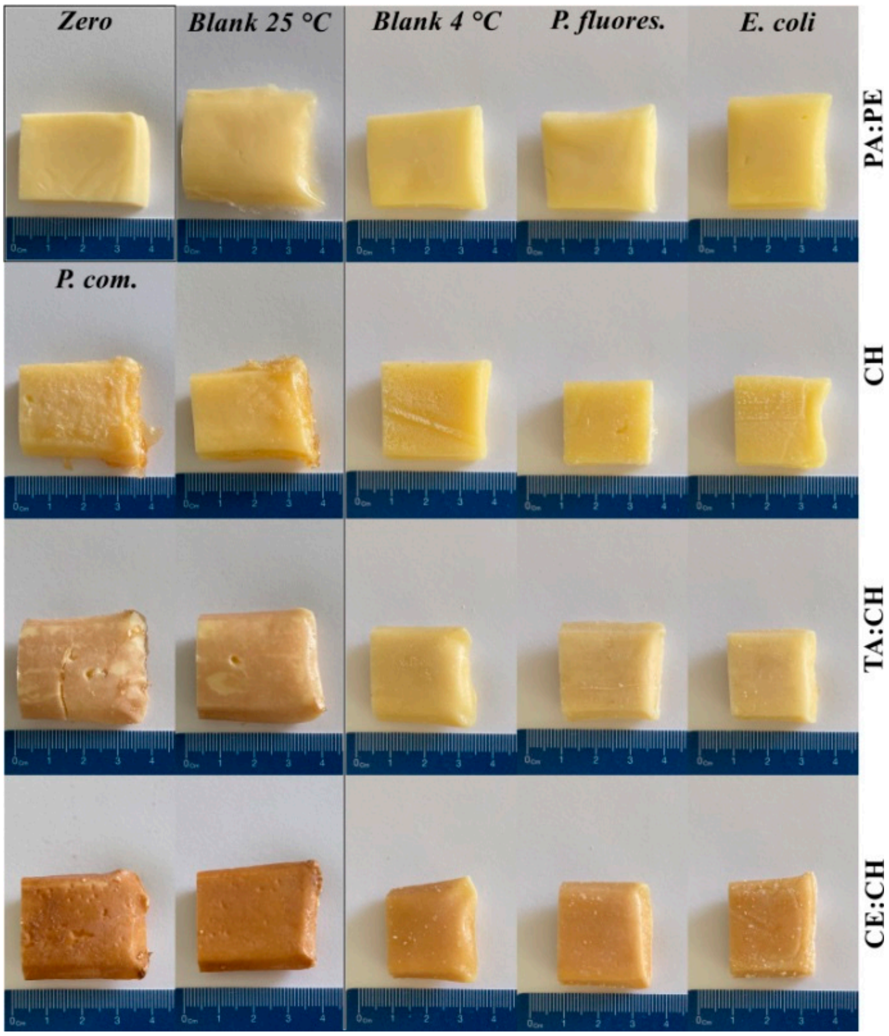

Figure S5. The appearance of the cheese at the beginning (Day 0) and the end of the storage (after 37 days, for all the other pictures).
